# 

**Published:** 1873-07

**Authors:** 


### Contents of July Number.

### ORIGINAL DEPARTMENT.

ART. I.—Case of Absence of the Uterus and Consequent Amenorrhœa, with Maltreatment of the patient. By Alfred T. Livingston, M. D. 441

ART. II.—Semi-Annual Meeting of the Erie County Medical Society, June 10th, 1873. Reported by D. E. Chace, M. D., Secretary...... 445

ART. III.—Abstract of Proceedings of the Buffalo Medical Association... 455

ART. IV.—Medical Society of the County of Albany, Semi-Annual Meeting June 10th. Reported by F. C. Curtis, M. D., Secretary........ 459

### MISCELLANEOUS.

The Treatment of Ulcers in Charity Hospital, New York................ 464

Ovariotomy.............................................. 465

The Treatment of Small Pox; Protective and Restorative.............. 466

The Process of Embalming.................................. 468

### EDITORIAL DEPARTMENT.

The Influence of Instruction on Public Health........................ 468

Massachusetts Medical Society and the Homeopathists................ 470

BOOKS REVIEWED:

Handbook for the Physiological Laboratory. By E. Klein and others.................................... 471

Books and Pamphlets Received.................................... 472

